# The genetics of overwintering performance in two-year old common carp and its relation to performance until market size

**DOI:** 10.1371/journal.pone.0191624

**Published:** 2018-01-25

**Authors:** Martin Prchal, Antti Kause, Marc Vandeputte, David Gela, Jean-Michel Allamellou, Girish Kumar, Anastasia Bestin, Jérôme Bugeon, Jinfeng Zhao, Martin Kocour

**Affiliations:** 1 University of South Bohemia in České Budějovice, Faculty of Fisheries and Protection of Waters, South Bohemian Research Center of Aquaculture and Biodiversity of Hydrocenoses, Vodňany, Czech Republic; 2 Natural Resources Institute Finland, Jokioinen, Finland; 3 GABI, INRA, AgroParisTech, Université Paris-Saclay, Jouy-en-Josas, France; 4 Ifremer, Palavas-les-Flots, France; 5 LABOGENA-DNA, Jouy-en-Josas, France; 6 SYSAAF, Rennes, France; Xiamen University, CHINA

## Abstract

Using farmed common carp, we investigated the genetic background of the second year overwintering performance and its relation to the performance during the third growing season and at market size. The experimental stock was established by partial factorial design with a series of 4 factorial matings of 5 dams and 10 sires each. The families were reared communally and pedigree was re-constructed with 93.6% success using 12 microsatellites on 2008 offspring. Three successive recordings (second autumn, third spring, and third autumn—market size) covering two periods (second overwintering, third growing season) were included. Body weight, Fulton’s condition factor and percent muscle fat content were recorded at all times and headless carcass yield and fillet yield were recorded at market size. Specific growth rate, absolute and relative fat change and overall survival were calculated for each period. Heritability estimates were significantly different from zero and almost all traits were moderately to highly heritable (*h*^*2*^ = 0.36–1.00), except survival in both periods and fat change (both patterns) during overwintering (*h*^*2*^ = 0.12–0.15). Genetic and phenotypic correlations imply that selection against weight loss and fat loss during overwintering is expected to lead to a better winter survival, together with a positive effect on growth in the third growing season. Interestingly, higher muscle fat content was genetically correlated to lower survival in the following period (*r*_g_ = -0.59; -0.53, respectively for winter and the third summer). On the other hand, higher muscle fat was also genetically linked to better slaughter yields. Moreover, selection for higher condition factor would lead to better performance during winter, growing season and at market size.

## Introduction

The aquaculture sector is among the fastest growing agricultural industries, as an increasing demand for fish cannot be supplied with stagnating capture fisheries. Genetic improvement of cultured stocks is playing an important role in optimizing and increasing aquaculture production [1].

Common carp (*Cyprinus carpio* and *Cyprinus rubrofuscus*) is one of the most cultured fish species in the world. Its aquaculture production is still increasing and reached over 4 million tons worldwide and 145 thousand tons in Europe in 2014 [2]. In the recent past, many studies concerning genetic improvement in common carp have been carried out [3–8]. However, most studies have been focused on production traits such as growth and yields. Yet, little is known about the genetic background of traits related to overwintering performance in common carp and about the impact of overwintering performance on the performance in the subsequent growing season.

Overwintering of common carp in temperate climate is considered a critical period with the risk of heavy loss of valuable fish. When water temperature is below 8°C, common carp significantly reduce their metabolism, decrease feed intake and lose weight [9,10]. Despite reduced metabolism, low feed intake results in utilizing of energetic reserves that become essential for winter survival [11]. In common carp lipid reserves are mobilized first [12], followed by glycogen and protein depletion [13,14] and as a result fish lose weight [9,13,15–17]. High losses of body weight and fast depletion of energetic reserves caused by a severe winter, suboptimal environmental conditions, predation, diseases, parasite assault, or combination of these factors lead to higher winter mortality [10]. The quantification of the impact of individual factors on winter mortality in a pond system is however hardly possible, and the impact of factors influencing fish survival varies over different conditions. Still, the survival of fish in a pond is a result of genetic and environmental factors which operate in combination. Good physical condition of fish before winter is thought to be important for survival, and may be represented with various traits. Therefore, the estimation of genetic parameters of such traits can help to understand their importance for winter survival.

After the overwintering period and the associated feed deprivation, re-feeding results in rapid recovery with catching-up growth and an increase in body fat [18,19]. Therefore, the impact of re-feeding and its genetic and/or phenotypic relation to traits at market size could also be of importance.

Survival is also a major economic trait related to performance over all rearing periods in common carp cultured under pond conditions. The overall survival of a stock may be seen as an indicator showing the ability of organisms to deal with environmental conditions. Until recently, nothing was known about genetic variance of survival in common carp. However, two studies confirmed that the heritability of survival is low to medium but significant [6,8]. Other important traits such as body weight, fat content, condition factor and slaughter yields showed moderate to high heritability [3–8], and thus selection seems to be a valuable tool for their improvement. However, data about genetic variance in dynamics of growth, muscle fat, survival and their correlations with other traits are scarce.

The aim of this study was to estimate the phenotypic and genetic parameters related to the second overwintering in common carp and to the third growing period at the end of which the fish reached market size. The intent was to i) reveal relationships among various traits which may affect the winter performance, ii) see how the winter performance is associated with the performance of fish during the third growing season and at market size and iii) estimate how breeding programs focused on various traits could affect winter survival or performance at market size in a carp breed.

## Materials and methods

### Ethics statement

The methodological protocol of the current study was approved by the expert committee of the Institutional Animal Care and Use Committee (IACUC) of the University of South Bohemia (USB) in České Budějovice, Faculty of Fisheries and Protection of Waters (FFPW) in Vodňany according to the law on the protection of animals against cruelty (Act no. 246/1992 Coll., ref. number 16OZ15759/2013-17214). The study did not involve endangered or protected species. The experimental stock was reared under the common semi-intensive pond management with regular checks (three times a week) of fish health and behavior. The experiment, from individual tagging to market size, ran from April 2015 to November 2016 (second and third growing seasons—GS and second winter period—W). At the end the fish were euthanized (humane endpoint) for evaluation of slaughter yields. The standard survival rates in common carp cultured in ponds are 60–80% during 2^nd^ GS, 80–95% during 2^nd^ W and 85–95% during 3^rd^ GS [20]. As we needed to have at least 1500 fish at the end of the experiment to ensure reliability of genetic parameters, 3000 fish were taken initially. At the end of the experiment, 1622 fish assigned to a single parental pair were euthanized for data collection. The observed survival rates were 67% during 2^nd^ GS, 98% during 2^nd^ W and 89% during the 3^rd^ GS. The total mortality for the whole period was lower than expected by Horváth et al. [20] and statistics of the Klatovy Fish Farm. The causes of mortality are likely multiple, including natural stress effects (fasting due to low temperatures, naturally occurring parasites) and predation, typical in the traditional ponds. To enhance animal welfare and decrease suffering during all fish handling, the fish were anaesthetized using 2-phenoxyethanol for each live trait recording, and humanely euthanized for final processing. The main author of study owns the certificate (CZ 01704) giving capacity to conduct and manage experiments involving animals according to section 15d paragraph 3 of Act no. 246/1992 Coll.

### Establishment of experimental stock

Artificial spawning of common carp was carried out at the hatchery of USB, FFPW, in Vodňany, Czech Republic. The broodstock fish were Amur mirror carp (Vodňany line), recently accepted as a new Czech common carp breed [21]. The Amur mirror carp are derived from the 2^nd^ generation of intercross of European mirror carp, *Cyprinus carpio*, and Amur wild (scaled) carp, *Cyprinus rubrofuscus*, selected to fix the homozygous “mirror” scale cover while incorporating genetic variation from the Amur wild carp. Presently, this new breed is used in crossbreeding programs and the commercial crossbreds perform 20–40% better in survival and growth compared to others [22]. Moreover, the breed, and even its crossbreds, displayed higher resistance to Koi herpes virus (KHV) infection than standard European carp breeds [23].

In March 2014, the pond with Amur mirror carp broodstock was drained and the available mature fish were transferred and kept in two ponds of 0.2 ha, one for each sex. In May 2014, the broodstock was checked again and fish in a good pre-spawning condition (evaluated by eye and hand inspection) were transferred to the hatchery and kept in tanks separated by sex at water temperature of 18°C. Fin tissue from caudal fin (approx. 1 cm^2^) was collected from each potential parent and stored in Eppendorf tube filled in with 98% ethanol at room temperature.

Artificial spawning of the broodstock fish was performed using the same protocol as described by Vandeputte et al. [3] at a water temperature of 21°C. Fish gametes were individually stored for a short time until mating. Sperm from 40 males was stored on ice in 200 ml cell culture containers, while stripped eggs from 20 females were stored at the hatchery temperature in dishes covered with foil.

A partial factorial design with a 4 series of mating designs of 5 dams and 10 sires each were used to produce experimental families. For each series, 100 grams of eggs from each dam were taken and pooled into one dish. The pooled eggs were then divided into 10 equal batches of 50 g of eggs and transferred into 10 cups of 200 ml. The cups with eggs were placed on an orbital agitator and inseminated individually by the sperm of a male. Fertilization was done by adding 50 ml of hatchery water while mixing the sperm and eggs with constant 200 rpm rotation speed with deflection 10 mm. One minute later, all the cups within each series were pooled to one dish and egg stickiness was eliminated with a milk solution. The process of fertilization was repeated four times, one per series. The duration between the first and the fourth series was less than two hours. At the end of all four mating series, the eggs from each mating were incubated in separate Zuger jars. After hatching, the yolk-sac fry from each Zuger jar were transferred and nursed in separate post-hatching incubators until swimming stage.

### Rearing of experimental stock and phenotypic recordings

At swimming stage the experimental stock was created by pooling equal quantities (estimated volumetrically) of larvae from all four post-hatching incubators. Larvae were transferred in plastic bags under oxygen atmosphere into prepared nursery ponds at USB FFPW and Klatovy Fish Farm (size of ponds 0.2–1 ha, stocking density 150,000 larvae. ha^-1^). The progenies were reared under semi-intensive pond conditions and the fish were feeding on natural food (plankton, benthos) and on additional pelleted feed [3] through the first growing season and first wintering until March 2015. Then, ponds were harvested and the pond with the best fish survival (50% survival, mean weight 15.8 ± 4.7 g) was taken for the next steps of the experiment. The fish were transferred to two tanks at USB FFPW facility in Vodňany. A random sample of 3000 fish was anesthetized with 2-phenoxyethanol (dose of 0.5 ml per 1 l of water) and then individually PIT-tagged and fin-clipped for further DNA extraction and genotyping in order to assign the fish to their parents. Each tagged individual was weighed (to the nearest 0.01 g) and measured for standard length (to the nearest mm).

In April 2015, all the tagged fish together with a reserve group of untagged fish from the same stock were transferred into an 1 ha pond (stocking density 6000 fish. ha^-1^) at Klatovy Fish Farm and reared for the second growing season the same way as during the first growing season.

In October 2015, the fish were harvested again. All survived fish (including a total of 2008 PIT tagged individuals) were transferred to tanks in the USB FFPW facility to be measured. Each tagged individual was identified with a tag reader and recorded for body weight (BW_1_) and standard length (SL_1_). Because of high genetic and phenotypic correlations between SL and BW at that stage, only BW was included in the further analysis. Fulton’s condition factor (FC) was calculated as FC_1_ = 10^5^ * BW_1_ / SL_1_^3^. Fat content in muscle (% Fat_1_) was measured using a Fish Fatmeter FM 692 (Distell Ltd., UK), using calibration CARP– 1. The fat percentage for each individual was calculated as the mean of four repeated measurements on the left side of the fish performed according to the manufacturer’s guidelines. The fat content measurements were performed by the same person during the whole experiment. To validate the accuracy of the muscle fat measurements, the values from Distell Fatmeter were compared with those analysed with sulpho-phospho-vanillin method [24] in 100 randomly sampled market-size fish. For the chemical fat analysis, the whole fillet with skin from each fish were homogenized using a mixer. The correlation between the Distell Fatmeter values and the chemically analysed values was 0.85.

After measuring of the PIT tagged individuals, they were stocked for overwintering in a 0.2 ha pond at USB FFPW pond facility in Vodňany. During winter, when water temperature is below 8°C, the fish radically reduce their metabolism, movement, and feed intake, and thus they are not fed with any additional food. However, during the experiment the winter conditions were mild. Average water temperature between November and March was 4.3°C, significant ice cover stayed for two weeks only and water temperature even increased above 8°C in November (two weeks) and February (four days). That is why altogether 75 kg of pelleted food was distributed to the pond during wintering in order to help the fish to stay in a good condition.

In March 2016, the pond was harvested and all survivors (*n* = 1976) were transferred into indoor tanks for data recording. The same traits as before the overwintering were recorded, BW_2_, SL_2_, FC_2_, and % Fat_2_. Furthermore, overwintering performance traits were calculated as follows: i) body weight change during wintering expressed as specific growth rate, SGR_1-2_ = (ln*w*_*t*_*−*ln*w*_0_)/*t*^-1^ * 100, where *w*_*t*_ is the final body weight (g), *w*_0_ is the initial body weight (g) and *t* is the duration of growth period in days, ii) absolute fat change, FatCh_1-2_ = Fat_2_ –Fat_1_, where Fat_2_ is the percent fat content after wintering and Fat_1_ is the percent fat content before wintering, iii) relative fat change, % FatCh_1-2_ = (Fat2 –Fat_1_) / Fat_1_ * 100, and iv) survival, during overwintering, Surv_1-2_, with 1 given for survived fish and 0 to fish not found during the trait recording.

In April 2016, the tagged fish were stocked in a 4-ha pond at Klatovy Fish Farm for the third growing season. Market-size fish were harvested in October 2016 and transferred into a storage pond in Vodňany and kept there for three weeks. This reflects the common commercial practice to empty the intestines and to refresh the odour and taste of the flesh [17,25]. Final recording was performed at the fish slaughter house of USB FFPW in České Budějovice, Czech Republic. In total 1622 fish with a single parental assignment were dressed out in November 2016. The fish were killed by a hit on the head and bled by cutting the gills according to the local rules. Standard length (SL_3_) was measured to the nearest 0.1 mm with an in-house electronic ruler. Fish were weighed (BW_3_) to the nearest 0.5 g, and muscle fat content (% Fat_3_) was recorded using the Distell Fish Fat Meter as described above. Subsequently, the fish were gutted, one fillet detached, sexed by visual inspection of gonads (females, males, immature) and each part of the processed body (head, fillet, viscera, gonads, skin, half carcass, ribs, fins, scales) was weighed to the nearest 0.5 g. Percentage of processed body [5] or so-called headless carcass yield (% hl-Carss) and fillet yield (% Fill) were calculated as the most important slaughter traits: % hl-Carss = (fillet + skin + trimmings + ribs + half carcass) / body weight * 100); % Fill = (fillet + skin) * 2 / body weight * 100. In addition, similar to the overwintering period FC_3_, SGR_2-3_, FatCh_2-3_, % FatCh_2-3_, and Surv_2-3_ were calculated for the growing period from spring to autumn. The fish with a visible deformity (*n* = 35) were excluded from further analysis.

### Parentage assignment

The fins tissue of parents and the experimental progeny (0.2 cm^2^) were placed into 96 well plates and sent to LABOGENA-DNA, the French laboratory for livestock genotyping (ISO 170025 accredited, Jouy-en-Josas, France). The parentage assignment was based on the analysis of 12 microsatellite loci (CCE46, HLJE265, HLJ2241, HLJ2346, HLJ2382, HLJ24657, HLJ2544, HLJ334, HLJ526, HLJ534, J58, and KOI 57–58). The parentage allocation was performed using AccurAssign software, applying a maximum-likelihood method [26]. The individuals without assignment to a single parental pair were discarded from further analysis (*n* = 129 fish).

### Quantitative genetic analysis

All trait values were checked for outliers that might indicate errors during measurements and recordings. Phenotypic (*V*_P_) and genetic variances (*V*_A_) and correlations (*r*_p,_ and *r*_g_, respectively) were estimated using DMU software [27]. The phenotypic variance (*V*_P_*)* was taken as the sum of all of the variance components as follows: *V*_P_ = *V*_A_ + *V*_D_ + *V*_R_, where *V*_D_ is the non-genetic maternal (dam) variance, and *V*_R_ is the residual variance. The software analyses data in multivariate mixed models using the restricted maximum likelihood method [28]. The genetic parameters were estimated using the following animal model:

$$Y_{ijkl}=\mu_{i}+{sex}_{j}+dam_{ik}+anim_{il}+e_{ijkl}$$

Where *Y*_*ijkl*_ is the vector of observations (for all analysed traits), *µ*_*i*_ is the overall mean for trait *i*, *sex*_*j*_ is the fixed effect for sex (*j* = female, male, unidentified), *dam*_*ik*_ is the random maternal effect of dam *k* for trait *i*, *anim*_*il*_ is the random genetic effect of an animal *l* (*l* = 1, 2, etc.–no. of individual) for trait *i*, and *e*_*ijkl*_ is the random residual. Models with and without the maternal effect were used to specifically test the effect of the model on heritability estimates. Genetic correlations were estimated without the dam effect because in most cases the maternal effect was negligible. Heritability estimated without the maternal effect was calculated as *h*^2^ = *V*_A_ / (*V*_A_ + *V*_R_) and with the maternal effect as *h*^2^ = *V*_A_ / (*V*_A_ + *V*_D_ + *V*_R_). The maternal effect was calculated as *m*^2^ = *V*_D_ / (*V*_A_ + *V*_D_ + *V*_R_). Heritability for survival was estimated on the observed binary scale and subsequently transformed to the underlying normally distributed liability scale using the formula by Dempster and Lerner [29]. Furthermore, residual covariance between a survival trait and the traits recorded at or after the survival trait recording was set to zero. Therefore, phenotypic correlations between these traits were not applicable.

## Results

### Parentage assignment

From the total of 2008 genotyped fish, 1879 fish (93.6%) were successfully assigned to a single parental pair, 96 (4.8%) were assigned to multiple parent pairs, and 33 (1.6%) were not assigned to any parental pair. Assigned fish (single parental pair) belonged to 199 out of the possible 200 full-sib families. The number of progeny per sire varied from 18 to 98, the average was 47. The number of progeny per dam varied from 27 to 160, the average was 94. The parentage assignment results were solid and similar to those achieved in other studies on common carp [3–5,7].

### Descriptive statistics of traits

Three successive recordings (before second wintering, after second wintering = before third growing season, and after third growing season) covering two periods (second overwintering, third growing season) were studied (Table 1). During overwintering, only a slight decrease in muscle fat content (FatCh_1-2_, % FatCh_1-2_), and surprisingly, a slight increase in weight were observed. The overwintering survival was high (98%). The growing period was expressed with rapid (recovery) growth (SGR_2-3_) and increasing fat content (FatCh_2-3_, % FatCh_2-3_). At market size, the *CV* for body weight was lower compared to the previous periods (14.6% vs 19.2 and 19.7%). The yields of hl-carcass (66%) and fillets (50%) were higher than usual in common carp, probably due to the specific dress out process which was different from the commercial one but reflected better the biological values of the traits. The level of Surv_2-3_ (89%) was typical for this age category and climatic conditions.

**Table 1:** Number of observations (*n*), traits means (mean ± S.D.), *CV* (coefficient of variation), *V*_P_ (phenotypic variance), *V*_A_ (genetic variance), *h*^2^ (heritability estimates ± S.E.), *m*^2^ (maternal effect ± S.E.) for traits within each studied period (_1_ before winter period_, 2_ after winter period_, 3_ at harvest) and for traits changes during _1–2_ overwintering period and _2–3_ growing period.

Trait	*N*	Mean ± S.D.	*CV*	*V*_P_	*V*_A_	*h*^2^ ± S.E.	*m*^2^ ± S.E.
**BW**_**1**_	1847	336.1 ± 64.6	19.2	4124.5	2022.1	0.49 ± 0.08	0.05 ± 0.07
**BW**_**2**_	1814	340 ± 67.1	19.7	4508.5	2307.4	0.51 ± 0.08	0.04 ± .0.07
**FC**_**1**_	1847	2.85 ± 0.25	8.8	0.0644	0.0468	0.73 ± 0.10	0.03 ± 0.09
**FC**_**2**_	1813	2.84 ± 0.24	8.5	0.0632	0.0598	0.93 ± 0.10	0.03 ± 0.11
**% Fat1**	1847	4.94 ± 1.26	25.5	1.62	1.01	0.62 ± 0.12	0.00 ± 0.08
**% Fat2**	1814	4.35 ± 1.11	25.5	1.26	0.45	0.64 ± 0.14	0.00 ± 0.08
**SGR**_**1-2**_	1814	0.004 ± 0.02	N/A	0.34	0.16	0.47 ± 0.11	0.00 ± 0.06
**FatCh**_**1-2**_	1813	-0.59 ± 0.64	108.5	0.41	0.052	0.12 ± 0.05	0.00 ± 0.02
**% FatCh**_**1-2**_	1814	-11 ± 12.8	116.4	164.64	21.42	0.13 ± 0.05	0.00 ± 0.02
**Surv**_**1-2(Obs)**_	1814	0.98 ± 0.16	N/A	0.0250	0.0004	0.02 ± 0.01	0.00 ± 0.03
**Surv**_**1-2 (Lia)**_	1814	N/A	N/A	N/A	N/A	0.13 ± 0.06^1^	0.00 ± 0.03
**BW**_**3**_	1559	1910 ± 279	14.6	80835.7	50873.1	0.63 ± 0.12	0.00 ± 0.08
**FC**_**3**_	1558	3.40 ± 0.32	9.4	0.0990	0.0986	1.00 ± 0.09	0.05 ± 0.12
**% Fat**_**3**_	1559	11.56 ± 2.96	25.6	8.40	5.67	0.67 ± 0.13	0.00 ± 0.09
**SGR**_**2-3**_	1559	0.89 ± 0.07	7.9	0.48	0.24	0.49 ± 0.10	0.00 ± 0.06
**FatCh**_**2-3**_	1557	7.24 ± 2.56	35.4	6.18	3.45	0.56 ± 0.10	0.00 ± 0.06
**% FatCh**_**2-3**_	1557	175 ± 72	41.1	5156.29	2398.84	0.47 ± 0.10	0.00 ± 0.06
**Surv**_**2-3 (Obs)**_	1622	0.89 ± 0.35	N/A	0.12	0.007	0.06 ± 0.02	0.00 ± 0.02
**Surv**_**2-3 (Lia)**_	1622	N/A	N/A	N/A	N/A	0.15 ± 0.05^1^	0.00 ± 0.02
**% hl-Carss**	1559	66.21 ± 2.19	3.3	3.83	1.36	0.36 ± 0.08	0.00 ± 0.05
**% Fill**	1559	49.75 ± 1.95	3.9	3.43	1.23	0.36 ± 0.08	0.00 ± 0.05

BW_1_ –BW_3_ = body weight, FC_1_ –FC_3_ = Fulton’s condition factor, % Fat_1_ –% Fat_3_ = muscle fat percent, % hl-Carss = headless-carcass yield, % Fill = fillet yield. Indices: 1 = the trait was recorded before wintering, 2 = the trait was recorded after wintering (before the third growing season), 3 = the trait was recorded after the third growing season (at market size). Overwintering period: SGR_1-2_ specific growth rate, FatCh_1-2_ = absolute fat change, % FatCh_1-2_ = relative fat change %, Surv_1-2(Obs)_ = overall survival on observed scale, Surv_1-2 (Lia)_ = overall survival on liability scale. Growing period: SGR_2-3_ specific growth rate, FatCh_2-3_ = absolute fat change, % FatCh_2-3_ = relative fat change %, Surv_2-3 (Obs)_ = overall survival on observed scale, Surv_2-3 (Lia)_ = overall survival on liability scale. N/A = not applicable.

^1^ = *h*^2^ ± S.E. was transformed to the liability scale using the formula by Dempster and Lerner [29].

### Heritability estimates

All the estimated heritabilities were significantly different from zero and almost all traits were medium to highly heritable (0.36–0.68) (Table 1). Surprisingly, the Fulton’s condition factors (FC1 –FC_3_) achieved heritability estimates from high to close to unity (0.73–1.0). Low heritability estimates were observed for FatCh_1-2_ and % FatCh_1-2_ (0.12–0.13). The heritabilities for survival were low for both periods (overwintering and growing season) and for both kind of estimations, on the observed scale (Surv_1-2obs_ = 0.02, Surv_2-3obs_ = 0.06) and on the underlying liability scale (Surv_1-2lia_ = 0.13, Surv_2-3lia_ = 0.15). The maternal effects *m*^2^ for all traits were insignificant and close to zero (<0.05).

### Genetic and phenotypic correlations

#### Correlations of traits related to overwintering

Genetic and phenotypic correlations among traits during overwintering are presented in Table 2. High (values in range 0.51–0.80) to strong (values > 0.80) phenotypic (0.68–0.98) and genetic correlations (0.98 for all traits) were observed for the same trait recorded before and after overwintering (i.e. BW1 –BW_2_, % Fat_1_ - % Fat_2_ and FC_1_ –FC_2_; S1 Table).

**Table 2:** Genetic (above the diagonal; ± S.E.) and phenotypic correlations (below the diagonal) of traits before wintering and traits changes during overwintering.

	BW_1_	SGR_1-2_	FC_1_	% Fat_1_	FatCh_1-2_	% FatCh_1-2_	Surv_1-2_
**BW**_**1**_	x	0.27 ± 0.14	0.08 ± 0.14	0.32 ± 0.13	-0.10 ± 0.18	0.05 ± 0.18	0.19 ± 0.31
**SGR**_**1-2**_	0.11	x	0.50 ± 0.11	-0.39 ± 0.16^1^	0.63 ± 0.12	0.67 ± 0.15	0.47 ± 0.29
**FC**_**1**_	0.06	0.14	x	-0.25 ± 0.14	0.21 ± 0.17	0.22 ± 0.16	0.26 ± 0.28
**% Fat**_**1**_	0.28	-0.14	0.00	x	-0.51 ± 0.14	-0.16 ± 0.17	-0.59 ± 0.26^2^
**FatCh**_**1-2**_	-0.05	0.16	-0.01	-0.46	x	0.92 ± 0.04	0.68 ± 0.30
**% FatCh**_**1-2**_	-0.01	0.29	0.00	-0.29	0.93	x	0.46 ± 0.33
**Surv**_**1-2**_	0.02	N/A	0.03	-0.01	N/A	N/A	x

See Table 1 for trait abbreviations. When covariate of body weight to % muscle fat content was used

^1^*r*_g_ = -0.47 ± 0.12

^2^*r*_g_ = -0.68 ± 0.28. N/A = not applicable.

Low positive phenotypic correlations were observed between BW_1_ and % Fat_1_ (*r*_p_ = 0.28) and between SGR_1-2_ and % FatCh_1-2_ (*r*_p_ = 0.29). A low negative correlation was estimated between % Fat_1_ and % FatCh_1-2_ (*r*_p_ = -0.29) and a moderate one was found between % Fat_1_ and FatCh_1-2_ (*r*_p_ = -0.46). A high positive phenotypic correlation was observed between FatCh_1-2_ and % FatCh_1-2_ (*r*_p_ = 0.93).

Regarding the genetic correlations between different traits, a strong positive genetic correlation was estimated between FatCh_1-2_ and % FatCh_1-2_ (*r*_g_ = 0.92 ± 0.04). High positive genetic correlations were estimated between SGR_1-2_ and the fat change traits, i.e. FatCh_1-2_ (*r*_g_ = 0.63 ± 0.12) and % FatCh_1-2_ (*r*_g_ = 0.67 ± 0.15), and also between FatCh_1-2_ and Surv_1-2_ (*r*_g_ = 0.68 ± 0.30), showing that several overwintering traits are related to each other. High negative genetic correlations were observed between % Fat_1_ and FatCh_1-2_ (*r*_g_ = -0.51 ± 0.14) and interestingly also between % Fat_1_ and Surv_1-2_ (*r*_g_ = -0.59 ± 0.26), indicating a link between reduced winter survival and high fat before winter. A moderate positive genetic correlation was estimated between BW_1_ and % Fat_1_ (*r*_g_ = 0.32 ± 0.13) and between SGR_1-2_ and FC_1_ (*r*_g_ = 0.50 ± 0.11). A moderate negative genetic correlation was observed between % Fat_1_ and SGR_1-2_ (*r*_g_ = -0.39 ± 0.16). To ensure that the negative relationships of % Fat_1_ with Surv_1-2_, FatCh_1-2,_ % FatCh_1-2_ and SGR_1-2_ were not generated by the relation of % Fat_1_ with BW_1_, the analysis was also run using BW_1_ as a covariate for % Fat_1_. With such a model, the genetic correlations become either more negative (SGR_1-2_ and Surv_1-2_) (Table 2.), or remain the same (FatCh_1-2_ and % FatCh_1-2_). Among the other pairs of traits, no significant genetic correlations were observed.

#### Correlations between traits related to overwintering and traits related to the third growing season

Estimated correlations among overwintering traits and traits related to the third growing season are listed in Table 3. When looking at the same traits between periods (before the third growing season and after the growing season) strong positive correlations (phenotypic as well as genetic) were observed for body weight, FC and muscle fat content (*r*_g_ = 0.70–0.94, *r*_p_ = 0.52–0.73). For the other traits, the correlations were insignificant (SGR) or with negative pattern for phenotypic and genetic correlations (FatCh, % FatCh and Surv).

**Table 3:** Genetic (first line; ± S.E.) and phenotypic correlations (second line) of traits changes during overwintering and traits after overwintering (left hand side) related to traits changes during growing period and traits at market size (upper heading).

	BW_3_	SGR_2-3_	FC_3_	% Fat_3_	FatCh_2-3_	% FatCh_2-3_	Surv_2-3_	% hl-Carss	% Fill
**BW**_**2**_	0.74 ± 0.07,	-0.59 ± 0.10,	0.15 ± 0.14,	0.22 ± 0.14,	0.13 ± 0.15,	-0.14 ± 0.15,	-0.29 ± 0.22,	-0.19 ± 0.15,	0.03 ± 0.16,
	0.72	-0.62	0.10	0.24	0.15	-0.07	-0.05	0.06	0.19
**SGR**_**1-2**_	0.62 ± 0.10,	0.11 ± 0.16,	0.54 ± 0.11,	-0.35 ± 0.13,	-0.33 ± 0.14,	-0.19 ± 0.15,	0.31 ± 0.24,	-0.37 ± 0.14,	-0.29 ± 0.15,
	0.34	-0.06	0.22	-0.10	-0.09	-0.05	0.03	-0.05	-0.05
**FC**_**2**_	0.55 ± 0.10,	0.34 ± 0.13,	0.94 ± 0.02,	-0.27 ± 0.13,	-0.22 ± 0.14,	-0.05 ± 0.15,	0.45 ± 0.21,	-0.14 ± 0.15,	-0.16 ± 0.15,
	0.29	0.08	0.73	-0.10	-0.11	-0.08	0.04	-0.06	-0.04
**% Fat**_**2**_	-0.15 ± 0.15,	-0.54 ± 0.11,	-0.27 ± 0.13,	0.70 ± 0.08,	-0.42 ± 0.13,	-0.36 ± 0.13,	-0.53 ± 0.19,	0.15 ± 0.15,	0.23 ± 0.15,
	0.04	-0.33	-0.04	0.52	0.16	-0.46	-0.02	0.05	0.14
**FatCh**_**1-2**_	0.17 ± 0.18,	0.17 ± 0.18,	0.19 ± 0.17,	-0.50 ± 0.14,	-0.50 ± 0.14,	-0.26 ± 0.17,	0.32 ± 0.26,	-0.45 ± 0.16,	-0.38 ± 0.16,
	0.03	0.06	0.06	-0.11	-0.16	-0.17	-0.02	-0.08	-0.08
**% FatCh**_**1-2**_	0.24 ± 0.17,	0.05 ± 0.18,	0.18 ± 0.17,	-0.25 ± 0.17,	-0.34 ± 0.16,	-0.37 ± 0.16,	0.10 ± 0.27,	-0.38 ± 0.17,	-0.26 ± 0.18,
	0.04	0.01	0.06	0.03	-0.12	-0.25	-0.02	-0.08	-0.06
**Surv**_**1-2**_	0.56 ± 0.31,	0.18 ± 0.30,	0.40 ± 0.27,	-0.18 ± 0.30,	-0.02 ± 0.30,	0.18 ± 0.33,	0.58 ± 0.35,	0.34 ± 0.30,	0.27 ± 0.30,
	-0.07	-0.04	0.00	0.08	-0.03	-0.11	0.24	0.002	0.001

See Table 1 for trait abbreviations.

Generally, phenotypic correlations were in most cases lower than genetic correlations. Only 11 phenotypic correlations out of 63 investigated were higher than 0.20 of which only four were higher than 0.50. For genetic correlations, 24 values out of 63 were significant of which 12 were higher than 0.50, and three were 0.70 or higher.

When looking at correlations between different traits, body weight and muscle fat content before the third growing season (BW_2_; % Fat_2_) were negatively correlated with specific growth rate (SGR_2-3_) during the third growing season (*r*_g_ = -0.59, -0.54; *r*_p_ = -0.62; -0.33, respectively). So, the leaner and smaller fish were performing better and catching up their larger counterparts. However, positive genetic and phenotypic correlations between BW_2_ and BW_3_ (*r*_g_ = 0.74; *r*_p_ = 0.72) also indicate that selection for body weight before the third growing season may increase market weight.

SGR_1-2_ showed high genetic correlations with BW_3_ and FC_3_ (*r*_g_ = 0.62 ± 0.10 and 0.54 ± 0.11, respectively) but phenotypic correlations were twice lower (0.34, 0.22, respectively). Significant but moderate negative genetic correlations were observed for SGR_1-2_ with % Fat_3_, FatCh_2-3_, and % hl-Carss and at the edge of significance with % Fill.

FC_2_ was positively genetically correlated to BW_3_ and Surv_2-3_ (high relationship) and SGR_2-3_ (moderate relationship) and negatively weakly correlated to % Fat_3._ In all cases, the phenotypic correlations were much lower_._ The condition factor after winter period thus seems to be a good indicator of the genetic merit of fish for several production traits in the third growing season.

Muscle fat content before the third growing season (% Fat_2_), while significantly correlated, was only in negative relationships with the traits of the following growing season. The genetic correlation with Surv_2-3_ (-0.53 ± 0.19) was highly negative indicating that higher muscle lipid level after winter is related to lower survival in the third growing season. Moreover, there were moderate genetic correlations with FatCh_2-3_ and % FatCh_2-3_. The phenotypic correlations were low to moderate or not existing.

The genetic correlations of fat change traits (FatCh_1-2_ and % FatCh_1-2_) both showed similar negative patterns with traits of the next period (FatCh_2-3_, % FatCh_2-3_ and % hl-Carss). For FatCh_1-2_, the correlations were moderate, for % FatCh_1-2_ low or moderate, but always lower than for FatCh_1-2_. Moreover, FatCh_1-2_ was significantly correlated with % Fat_3_ (*r*_g_ = -0.50 ± 0.14) and with % Fill (*r*_g_ = -0.38 ± 0.16). Oppositely, % FatCh_1-2_ was significantly correlated with % FatCh_2-3_ (*r*_g_ = -0.37 ± 0.16). The phenotypic correlations were low or negligible.

Genetic and phenotypic correlation between Surv_1-2_ and the traits after the third growing season were not significant.

## Discussion

The present study focused on the genetic variance of the second winter performance and on the effect of overwintering traits on traits of the third growing season, at the end of which the fish reached the market size. The most important fish characteristics for winter performance are survival, but also a condition that ensures good recovery and performance of fish in the next rearing period. A trait that has often been mentioned to be important for winter survival is muscle fat content [12]. Complementary traits that may indicate either relationship to overwintering performance or recovery after the winter period, are weight change and fat change, which were in our study expressed as specific growth rate [30] and as absolute and relative fat change. Furthermore, Fulton’s condition coefficient is also often used in common carp culture as a trait indicating actual condition [9]. So, all these traits were evaluated for their importance for winter survival and performance until market size.

### Genetic and maternal variance

The heritability of traits in this study was estimated using two models: either including or excluding the random non-genetic maternal effect *m*^2^. The maternal effect for all the estimates (after second and third growing season) was negligible, similar to the studies by Vandeputte et al. [3] and Ninh et al. [7].

The heritability estimates for BW, % Fat, SGR, FatCh, % hl-Carss and % Fill were mostly moderate to high and tended to increase with the age of the fish. This observation was in accordance with other recent studies on common carp [3–8] in different breeds/strains and under various pond management conditions. Thus, common carp has sufficient genetic variance in most important performance traits (growth, fat and yield) for selective breeding programs. The results also show that in Central European climatic conditions, the selection of fish should be done optimally after the second wintering (S2 and S3 Tables). At this period the fish are still small enough for easy handling and short-term storage and there is a reasonably high genetic correlation (0.74) between the weight at this age and market-size weight.

Low but significant heritability estimates were found for survival during wintering as well as third growing season. Dong et al. [8] observed similar estimates for overall survival during four generations, while Nielsen et al. [6] observed heritability of 0.34 for survival during the last growing season. Similar variability was also reported in other fish species [31–37]. This is not surprising as reasons for fish mortality and the range of mortality differed across studies. Generally, survival has low *h*^2^ when low mortality rates are observed. This was also our case. However, the existing genetic variation for overall survival indicates that there is a potential for improving general robustness against various mortality factors [34].

Fulton’s condition factor was very highly heritable in all periods (from yearling to market size) and even close to unity at harvest (*h*^2^ = 0.997). Vandeputte et al. [3] estimated a much lower heritability for FC in juvenile common carp (0.37). Thus, in our study the FC variation was mostly genetic variance and environmental variance became negligible. Moreover, we found that genetic and phenotypic correlations among FC and biometrical indices (relative body height, relative body width) were strong (> 0.9; S4 Table). So, FC indicated also the shape of fish–fish with higher FC had higher and wider body. Similarly, a strong relationship between body shape and FC was observed in European whitefish, *Coregonus lavaretus* [36]. In our study, this phenomenon might be partly due to the fact that the great-grand parents of the experimental stock were very different in body shape (oblong-like body shape in Amur wild carp and square-like body shape in the maternal strain [21]). This generates high genetic variance in the F3 generation used in the present study.

### Overwintering performance

No significant genetic correlations were observed between winter survival (Surv_1-2_) and fish weight (BW_1_)_._ So, selection for higher body weight before winter would not lead to any favorable response in overwintering survival. Interestingly, despite generally high survival (98%), a significant negative genetic correlation between % Fat_1_ and Surv_1-2_ (-0.59) was observed. Hence, selecting for higher muscle fat could lead to lower winter survival. However, this observation might be specific only for the mild winter conditions that were experienced here. Such observations contradict the general assumption concerning size-selective mortality in fish–smaller fish tend to have lower energy reserves and use those reserves more rapidly due to the allometry of metabolic rate, which results in lower survival [12,38,39]. However, Biro et al. [39] observed in rainbow trout (*Oncorhynchus mykiss*) that the larger/fatter individuals unlike the smaller/leaner ones consumed more of their lipid reserves than predicted by standard metabolic allometry. We observed that heavier fish before winter were slightly fatter (*r*_p_ = 0.28) and that selection for heavier fish would lead to a slight increase in muscle fat content (*r*_g_ = 0.32). However, phenotypic correlations between % Fat_1_ and FatCh_1-2_ as well as % Fat_1_ and % FatCh_1-2_ were negative. So, it also shows that fatter fish mobilized their lipid reserves more than the leaner ones.

We assume that due to the mild winter conditions, fish were more active than normally, needed more energy and were looking for food. Otherwise, fish could not increase or keep their weight (decreased BW in 713 fish, no BW change in 67 fish, increased BW in 1034 fish). Similar foraging behavior in three-year old carp was observed by Bauer and Schlott [9] but in their study, all fish except one lost weight during winter, probably because of colder winter and absence of winter feeding. However, due to climate change, mild winter conditions might be more often expected in Europe in the future [40]. Former recommendations and our observation suggest that importance of parameters for survival might depend on e.g. the age of fish, food availability during winter and the climatic conditions [10].

Other results supporting a disadvantage of having too high muscle fat content prior to overwintering might be seen from the correlations among % Fat_1_, SGR_1-2_, FatCh_1-2_, % FatCh_1-2_ and Surv_1-2_. Selecting fish for higher muscle fat content before winter would lead to spending more muscle fat during winter in absolute value (moderate correlation), and to having lower SGR_1-2_. Moreover, if a selection on lower weight loss during winter was done, the fish would tend to be initially leaner (decreasing of % Fat_1_) and to have lower fat decrease during winter in absolute (FatCh_1-2_) as well as relative values (% FatCh_1-2_). Summing up, selection for lower decreases in weight and muscle fat content is expected to result in better winter performance. This observation is in accordance with assumptions by Schäperclaus [41], Bernard and Fox [42], and Pratt and Fox [43]. However, such selection would likely lead also to a decreasing of muscle fat content before wintering. This is contradictory to studies on European sea bass (*Dicentrarchus labrax*), where fish that lost less weight when fasting were also those that exhibited higher muscle fat content after starvation [44–46]. On the other hand, those fish were completely feed-deprived and this fact might be a reason for this opposite result, while in our case fish had a chance to forage. It might happen that during mild winter, natural selection would privilege leaner fish to perform better e.g. due to compulsion to ingest more feed and thus maintain their weight and lipid stores more effectively. The same strategy of leaner fish was also observed in Atlantic salmon (*Salmo salar* L.) by Johansen et al. [47]. Oppositely, fatter fish, not being forced to forage, would be handicapped during mild winter and due to higher metabolic activity they would lose more lipid stores and weight which would affect their survival. Thus, fish may be able to recognize their lipid reserves status, a capacity termed lipostatic regulation that was also reported in other fish species [18,47–52].

The condition factor before winter (FC_1_) was not phenotypically correlated to any trait, but selection for this trait should result in lower weight loss (or weight gain) during winter (*r*_g_ for FC_1_: SGR_1-2_ = 0.50) that might be advantageous for winter performance. However, due to mild winter, FC decreased only slightly during winter, even survival was high, and this could be reason why this trait was only slightly related to overwintering performance. Higher mortality during winter in fish with FC decrease exceeding 15–20% was observed in a previous study [53] and accordingly, FC was of interest when assessing winter survival in common carp [9].

### Impact of overwintering performance on the next growing season until market size

The conditions during the third growing season were optimal as seen from standard-level survival (89%), average water temperature from April to October (17.5°C), from the mean body weight gain of 1570 g, from the total pond production of 665 kg. ha^-1^, and from a considerable increase in muscle fat (abs: 7.2%), which were higher than usual.

Similar to overwintering period, we found that the muscle fat content after overwintering (% Fat_2_) was negatively genetically correlated (-0.53) to survival during the third growing season (Surv_2-3_). Conversely, there was no correlation between % Fat_2_ and BW_3._ Thus, selective breeding for restricted fat content in spring may increase survival without affecting final body weight. The negative correlation between muscle fat content and survival has no straightforward explanation. Kause et al. [36] observed in European whitefish a rather positive genetic correlation between higher fillet lipid at harvest and survival. However, in that case larger fish were also fatter than the smaller ones. While a certain level of muscle fat is essential for various biological functions of fish [54,55], our observations suggest that an excess might have disadvantageous effects.

The negative moderate to high phenotypic and high genetic correlations of BW_2_ and % Fat_2_ with SGR_2-3_ indicated that initially smaller and leaner fish grew faster during the third growing season. On the other hand, smaller fish did not catch in weight the larger ones at market size. Still, it looks that worse performing genotypes were supported after the period of growth depression by good rearing conditions, as described above, to catch up the other genotypes. This fact also decreased *CV* of body weight. Likewise, Mas-Muñoz et al. [56] observed negative phenotypic correlation between initial body weight and SGR in sole (*Solea solea*) grown in ponds.

The change of weight during overwintering (SGR_1-2_) was favorably genetically and phenotypically correlated to BW_3_ and FC_3_ (*r*_g_ = 0.62, 0.54; *r*_p_ = 0.34, 0.22, respectively). So, the fish which grew or lost less weight during overwintering achieved also higher body weight and condition at market size. Accordingly, selection for higher SGR_1-2_ (the lowest negative or positive value) could positively affect the body weight and condition at market size. On the other hand, selection for higher SGR_1-2_ would likely lead to a decrease of muscle fat content (% Fat_3_) and slaughter yields at market size (*r*_g_ = -0.29 and -0.35, respectively). The same situation regarding final muscle fat content and edible parts yield would happen when selecting on higher FatCh_1-2_ (lower decrease or slight increase in muscle fat content during winter). Thus, the correlations indicate that genotypes which tended to lose more muscle fat during winter compensated the muscle fat during growing season and the higher muscle fat content very likely increased hl-carcass and fillet yields in such fish (as estimated based on positive correlations between % Fat_3_ related to % hl-Carss and % Fill; S4 Table). Similarly, positive correlations between muscle fat content and slaughter yields were observed in rainbow trout [57] and previous study in common carp [5]. Hence, the possible decrease in slaughter yields makes the selection on SGR_1-2_ or FatCh_1-2_ less appealing. Nevertheless, the negative correlation between SGR_1-2_ or FatCh_1-2_ and yields might be overcome with a multitrait selection method, e.g. looking for predictors for better slaughter yields similarly as in rainbow trout [58,59] and European seabass [60]. However, in case that flesh quality were more profitable for fish farmers than increased dress-out yields, decreasing the fat in muscle would most likely lead to increasing relative rate of polyunsaturated fatty acids (PUFAs) and improving the omega 3: omega 6 fatty acids profile [61,62]. Then SGR_1-2_ or FatCh_1-2_ would become interesting traits for a selection program.

Selection for higher FC_2_ would lead to increasing final weight (BW_3_), SGR_2-3_ and Surv_2-3_ (*r*_g_ = 0.34–0.55) with a slight decrease in a final muscle fat content (*r*_g_ = -0.27) with no effect on slaughter yields. A high positive genetic correlation between FC and BW was found e.g. in rainbow trout by Haffray et al. [63], but oppositely Sae-Lim et al. [37] found no genetic correlation between fingerling FC and BW or survival at harvest. Similarly, correlations between FC and muscle fat content differ among studies [36]. In our study, FC_2_ after overwintering seemed to be quite a valuable trait for a potential selective breeding program in Amur mirror carp.

## Conclusions

Muscle fat content is a trait playing an important role in biological functions of common carp. In this study it was found that selection for i) lower fat content before and after winter, ii) lower decrease in muscle fat content and/or body weight during winter, may both lead to better survival and growth during the subsequent growing period. On the other hand, edible parts yield may slightly decrease. We also showed that selection for higher condition factor might result in better performance during the winter, and mainly during the third growing season and at market size. However, this would also lead to a change of fish conformations to a less favourable square-like body shape.

## Supporting information

S1 Table Genetic and phenotypic correlations of traits before and after second overwintering. (DOCX)

S2 Table Genetic correlations of body weight and Fulton’s condition factor in one-year old common carp related to traits (BW, FC, % Fat) during all recorded periods. (DOCX)

S3 Table Phenotypic correlations of body weight and Fulton’s condition factor in-one year old common carp related to traits (BW, FC, % Fat) during all recorded periods. (DOCX)

S4 Table Genetic and phenotypic correlations of selected traits at market size. (DOCX)
