# Correction: The genetics of overwintering performance in two-year old common carp and its relation to performance until market size

**DOI:** 10.1371/journal.pone.0197820

**Published:** 2018-05-17

**Authors:** The PLOS ONE Staff

The affiliation listed for Jérôme Bugeon is incorrect. The correct affiliation is: LPGP, INRA, Rennes, France.

There are errors in the Author Contributions. The publisher apologizes for these errors. Please view the correct contributions below.

**Conceptualization:** Antti Kause, Marc Vandeputte, Martin Kocour.

**Data curation:** Martin Prchal, Antti Kause.

**Formal analysis:** Martin Prchal, Antti Kause.

**Funding acquisition:** Antti Kause, Marc Vandeputte, Martin Kocour.

**Investigation:** Martin Prchal, Marc Vandeputte, David Gela, Jean-Michel Allamellou, Girish Kumar, Anastasia Bestin, Jérôme Bugeon, Jinfeng Zhao, Martin Kocour.

**Methodology:** Antti Kause, Marc Vandeputte, Martin Kocour.

**Project administration:** Antti Kause, Marc Vandeputte, Martin Kocour.

**Resources:** Antti Kause, Marc Vandeputte, David Gela, Jérôme Bugeon, Martin Kocour.

**Supervision:** Antti Kause, Marc Vandeputte, Martin Kocour.

**Validation:** Martin Prchal, Antti Kause, Marc Vandeputte, Martin Kocour.

**Visualization:** Martin Prchal, Antti Kause, Martin Kocour.

**Writing–original draft:** Martin Prchal, Antti Kause, Marc Vandeputte, Martin Kocour.

**Writing–review & editing:** Martin Prchal, Antti Kause, Marc Vandeputte, David Gela, Jean-Michel Allamellou, Girish Kumar, Anastasia Bestin, Jérôme Bugeon, Jinfeng Zhao, Martin Kocour.
