# Supplementary material for: The genetics of overwintering performance in two-year old common carp and its relation to performance until market size
Source: PLoS One. 2018 Jan 25;13(1):e0191624. doi: 10.1371/journal.pone.0191624 (PMC5784954; doi:10.1371/journal.pone.0191624)
Supplement: S1 Table — (DOCX) [file pone.0191624.s001.docx]

**S1 Table. Genetic (first value; ± S.E.) and phenotypic correlations (second value) of traits before (left hand side) and after second overwintering (upper heading).**

|  | **BW_2_** | **FC_2_** | **% Fat_2_** |
| --- | --- | --- | --- |
| **BW_1_** | 0.98 ± 0.01, 0.98 | 0.10 ± 0.15, 0.08 | 0.33 ± 0.13, 0.28 |
| **FC_1_** | 0.17 ± 0.14, 0.09 | 0.98 ± 0.01, 0.68 | -0.22 ± 0.14, -0.01 |
| **% Fat_1_** | 0.23 ± 0.14, 0.25 | -0.28 ± 0.13, -0.06 | 0.98 ± 0.01, 0.86 |

BW_1_ – BW_2_ = body weight, FC_1_ – FC_2_ = Fulton’s condition factor, % Fat_1_ – % Fat_2_ = muscle fat percent.
