# Supplementary material for: The genetics of overwintering performance in two-year old common carp and its relation to performance until market size
Source: PLoS One. 2018 Jan 25;13(1):e0191624. doi: 10.1371/journal.pone.0191624 (PMC5784954; doi:10.1371/journal.pone.0191624)
Supplement: S2 Table — (DOCX) [file pone.0191624.s002.docx]

**S2 Table. Genetic correlations (± S.E.) of body weight and Fulton’s condition factor in one-year old common carp (left hand side) related to traits (BW, FC, % Fat) during all recorded periods (upper heading).**

|  | **BW_1_** | **BW_2_** | **BW_3_** | **FC_1_** | **FC_2_** | **FC_3_** | **% Fat_1_** | **% Fat_2_** | **% Fat_3_** |
| --- | --- | --- | --- | --- | --- | --- | --- | --- | --- |
| **BW_0_** | 0.76±0.07 | 0.72±0.07 | 0.23±0.14 | -0.08±0.14 | -0.08±0.14 | -0.17±0.14 | 0.55±0.11 | 0.57±0.11 | 0.26±0.14 |
| **FC_0_** | -0.17±0.15 | -0.12±0.15 | 0.17±0.15 | 0.68±0.07 | 0.72±0.07 | 0.61±0.10 | -0.11±0.15 | -0.15±0.15 | -0.22±0.15 |

BW_0_ – BW_3_ = body weight, FC_0_ – FC_3_ = Fulton’s condition factor, % Fat_1_ – % Fat_3_ = muscle fat percent.

_0_ - at tagging (one-year old), _1_ – before second overwintering, _2_ – after second overwintering _3_ - at market size (three-year old).
