# Supplementary material for: The genetics of overwintering performance in two-year old common carp and its relation to performance until market size
Source: PLoS One. 2018 Jan 25;13(1):e0191624. doi: 10.1371/journal.pone.0191624 (PMC5784954; doi:10.1371/journal.pone.0191624)
Supplement: S3 Table — (DOCX) [file pone.0191624.s003.docx]

**S3 Table. Phenotypic correlations of body weight and Fulton’s condition factor in one-year old common carp (left hand side) related to traits (BW, FC, % Fat) during all recorded periods (upper heading).**

|  | **BW_1_** | **BW_2_** | **BW_3_** | **FC_1_** | **FC_2_** | **FC_3_** | **% Fat_1_** | **% Fat_2_** | **% Fat_3_** |
| --- | --- | --- | --- | --- | --- | --- | --- | --- | --- |
| **BW_0_** | 0.69 | 0.68 | 0.35 | -0.02 | -0.001 | -0.04 | 0.32 | 0.32 | 0.18 |
| **FC_0_** | -0.06 | -0.03 | 0.07 | 0.34 | 0.43 | 0.36 | -0.02 | -0.02 | -0.08 |

BW_0_ – BW_3_ = body weight, FC_0_ – FC_3_ = Fulton’s condition factor, % Fat_1_ – % Fat_3_ = muscle fat percent.

_0_ - at tagging (one-year old), _1_ – before second overwintering, _2_ – after second overwintering _3_ - at market size (three-year old).
