# Supplementary material for: The genetics of overwintering performance in two-year old common carp and its relation to performance until market size
Source: PLoS One. 2018 Jan 25;13(1):e0191624. doi: 10.1371/journal.pone.0191624 (PMC5784954; doi:10.1371/journal.pone.0191624)
Supplement: S4 Table — (DOCX) [file pone.0191624.s004.docx]

**S4 Table. Genetic (first value; ± S.E.) and phenotypic (second value) correlations of selected traits at market size.**

|  | **RBH_3_** | **RBW_3_** | **% hl-Carss** | **% Fill** |
| --- | --- | --- | --- | --- |
| **FC_3_** | 0.97 ± 0.01;0.89 | 0.94 ± 0.01; 0.75 | X | X |
| **% Fat_3_** | X | X | 0.32 ± 0.13, 0.26 | 0.37 ± 0.13, 0.34 |

Left hand side: FC_3_ = Fulton’s condition factor, % Fat_3_ = muscle fat percent.

Upper heading: RBH_3_ = relative body height, RBW_3_ = relative body width, % hl-Carss – headless-carcass yield, % Fill = fillet yield.
